# Engineering Stability: Cholesterol-Modulated Liposome Response to Physical and Chemical Stressors for Enhanced Antimicrobial Activity

**DOI:** 10.3390/ph19030366

**Published:** 2026-02-26

**Authors:** Luísa Morato Ribeiro, Cínthia Caetano Bonatto, Luciano Paulino Silva

**Affiliations:** 1 Postgraduate Program in Nanoscience and Nanobiotechnology, University of Brasília, Brasília 70910-900, DF, Brazil; luisamoraribe@gmail.com; 2 Laboratory of Nanobiotechnology (LNANO), Embrapa Genetic Resources and Biotechnology, Brasília 70770-917, DF, Brazil; cinthiabonatto@gmail.com

**Keywords:** liposomes, cholesterol, streptomycin, stress, microorganisms

## Abstract

**Background/Objectives**: Liposomes are promising carriers for enhancing antibiotic delivery, but their stability under various stress conditions is crucial for clinical applicability. **Methods**: This study aimed to evaluate the physicochemical and antimicrobial stability of streptomycin-loaded liposomes subjected to mechanical (bath ultrasonication, probe ultrasonication, ultra-turrax homogenization), thermal (freezing, heating), and chemical (H_2_O_2_, Triton X-100, sodium dodecyl sulfate—SDS) stressors. **Results**: Isolated mechanical stresses (probe ultrasonication, ultrasonic bath, and ultra-turrax) did not significantly affect hydrodynamic diameter (DH), polydispersity index (PdI), or Zeta potential (ZP) (*p* > 0.05). In contrast, combined ultrasound–freezing stress induced marked destabilization, with DH increasing from ~110 nm to 362 nm (Lc0uf) and from 109 nm to 334 nm (Lc1uf), accompanied by PdI increases from ~0.13 to 0.37–0.41 and a ZP shift in Lc1uf from −43.1 mV to −60.1 mV. Thermal exposure at 75 °C and freezing at −30 °C caused no significant changes in DH or PdI, whereas freezing at −80 °C led to severe destabilization, with over fourfold increases in DH and fivefold increases in PdI; the cholesterol-free formulation (Lc0t−80) reached ~664 nm and a PdI of 0.609. Chemical oxidation with 1% H_2_O_2_ did not affect DH, PdI, or ZP, while surfactants (1% Triton X-100 and 1% SDS) significantly altered PdI and ZP in a cholesterol-dependent manner. MIC assays showed that formulations stressed by freezing at −80 °C or by combined ultrasound–freezing retained activity against *Escherichia coli* (MIC = 50 µg/mL), whereas SDS abolished activity. For *Staphylococcus aureus*, all stressed formulations remained active, and SDS reduced the MIC from 12.5 to 5.625 µg/mL. **Conclusions**: These findings demonstrate the robustness and stress-specific stabilization of these liposomal formulations, confirming that the antibiotic’s activity is preserved, which highlights their potential for therapeutic use.

## 1. Introduction

Microbial resistance is defined as the ability of microorganisms to withstand the action of antimicrobial agents. Consequently, persistent infections occur, leading to increased morbidity and mortality rates, as well as higher healthcare costs associated with prolonged hospital treatments [1,2]. Furthermore, the slow progress in the development of new antibiotics intensifies this scenario, making microbial resistance one of the greatest global public health challenges and emphasizing the urgent need for alternative strategies to ensure antimicrobial efficacy [3,4].

In this context, nanocarriers have gained increasing attention as a promising strategy to overcome the limitations of conventional antibiotics and to reduce the likelihood of resistance selection [5]. Among the various nanocarriers, lipid-based nanostructured systems—particularly liposomes—have been extensively explored due to their versatility and favorable biocompatibility [6].

Liposomes are spherical vesicles composed of one or more phospholipid bilayers capable of encapsulating both hydrophilic and hydrophobic compounds [7]. Their structure mimics biological membranes, which confer cell fusion properties; moreover, they can encapsulate, protect, and release active compounds in a controlled manner. These characteristics make liposomes attractive candidates for the development of antibiotic drug delivery systems (DDS), particularly in the context of rising antimicrobial resistance [5,8].

Aminoglycoside antibiotics, such as streptomycin, have been widely used to treat infections caused by Gram-positive and Gram-negative bacteria due to their ability to inhibit bacterial protein synthesis [9]. However, their use is limited by adverse effects, including nephrotoxicity and ototoxicity, as well as by increasing bacterial resistance, which requires progressively higher therapeutic doses. The encapsulation of these drugs in liposomes thus emerges as a potential strategy to enhance antimicrobial activity while reducing associated toxicity [10,11].

Resistant bacteria can become susceptible to an antibiotic once it is encapsulated in liposomes. This effect has been associated with drug protection from enzymatic degradation, increased fusogenicity with the bacterial membrane [12], enhanced intracellular delivery, improved solubility and stability, and targeted delivery directly to the site of action, thereby potentially minimizing resistance selection [13,14,15]. However, the therapeutic efficacy of liposomes is intimately linked to physicochemical properties such as size, surface charge, and bilayer fluidity, which are primarily determined by the synthesis method and the components employed [16,17,18].

Among these components, cholesterol is commonly incorporated to increase stability by reducing phospholipid bilayer fluidity through intercalation among the lipid tails [13]. Furthermore, cholesterol can influence liposome fusogenicity with target cells, antibiotic release profile, encapsulation efficiency [19,20], and improve antimicrobial activity [21]. Therefore, understanding the modulatory role of cholesterol on liposomal properties is essential for optimizing their application in antibiotic DDS [8,22,23].

Despite these advantages, liposomes face stability challenges due to chemical degradation (e.g., hydrolysis and oxidation of phospholipid chains [22,23]) and physical degradation (e.g., aggregation, melting induced by mechanical stress, surfactants, temperature, pH, oxidation, or storage [24,25,26]). Recent advances in stimulus-responsive liposomes address this problem by leveraging external triggers such as temperature, ultrasound, oxidants, or detergents for controlled release, balancing stability with on-demand drug delivery [25,27,28,29]. For example, thermosensitive liposomes release payloads upon heating, while ultrasound-responsive systems disrupt bilayers through cavitation [30,31]. Oxidative stress, common in infected tissues, can trigger release in redox-sensitive formulations [32]. Detergents mimic endogenous surfactants, such as bile salts, altering membrane permeability [33]. These responsive designs have shown promise in antimicrobial applications, increasing penetration into biofilms and reducing off-target effects [34,35,36,37].

However, detailed characterization of liposomal responses to such stress remains limited, particularly regarding specific cholesterol modulation in response to stress. This study innovates by systematically evaluating streptomycin-loaded liposomes under mechanical, thermal, and chemical stresses, integrating physicochemical analyses (DH, PdI, ZP) with functional antimicrobial assays (MIC, MBC against *Escherichia coli* and *Staphylococcus aureus*). Unlike previous work that focused on formulation development or basic physicochemical characterization, our approach systematically correlates stress-induced physicochemical changes with biological activity, providing a more application-oriented understanding of how liposomal composition influences stability and functional performance under conditions relevant to pharmaceutical processing and storage.

## 2. Results

To systematically evaluate the stability of streptomycin-loaded liposomes, four distinct formulations were engineered with increasing cholesterol content (0, 1, 3, and 5 mg/mL), designated as Lc0, Lc1, Lc3, and Lc5, respectively. These formulations were subjected to a panel of ten distinct stress conditions, encompassing mechanical, thermal, chemical, and combined mechanical-thermal challenges, designed to simulate potential destabilizing events during production, storage, and handling. A comprehensive overview of all formulation codes and their corresponding stress treatments is provided in Table 1 and Supplementary Table S1. The following sections detail the effects of cholesterol on baseline physicochemical properties and the subsequent impact of each stress category on liposomal integrity and antimicrobial function.

### 2.1. Influence of Cholesterol on Physicochemical Properties

**Figure 1:**
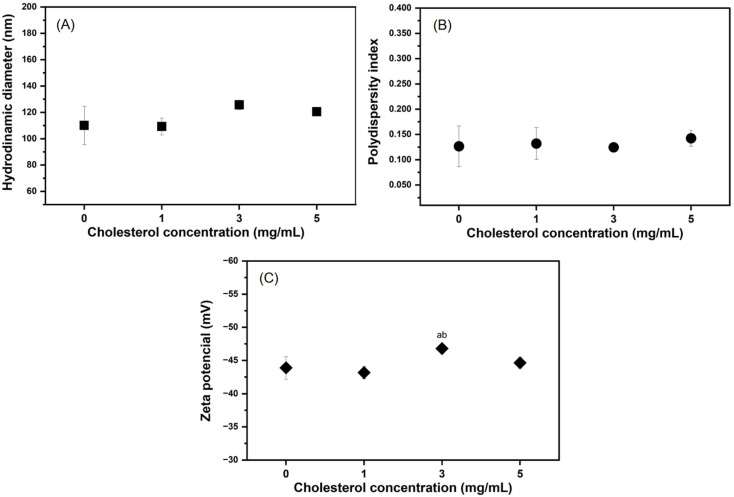
Effect of different cholesterol concentrations (0, 1, 3, and 5 mg/mL) on the physicochemical properties of liposomes. (**A**) Hydrodynamic diameter (DH), (**B**) polydispersity index (PdI), and (**C**) Zeta potential (ZP) of liposomes containing different cholesterol concentrations. Data represent mean ± standard deviation (SD) of three replicates (technical triplicates averaged for each replicate). No statistically significant differences were observed between cholesterol concentrations for the DH or PdI parameters (one-way ANOVA, *p* > 0.05), while differences were observed for ZP at 3 mg/mL when compared to 0 and 1 mg/mL, as indicated by lowercase letters a and b, respectively.

To investigate the influence of cholesterol on liposomal properties, formulations containing increasing concentrations of cholesterol (0, 1, 3, and 5 mg/mL) were prepared and characterized in terms of hydrodynamic diameter (DH), polydispersity index (PdI), and Zeta potential (ZP) (Figure 1, Supplementary Table S2, and Supplementary Figures S1 and S2). The results showed that the incorporation of cholesterol, even at the highest concentration (5 mg/mL), did not result in statistically significant changes (*p* > 0.05) in any of the physicochemical parameters evaluated when compared to the cholesterol-free formulations (Figure 1), with the exception of the Zeta potential (ZP) observed for Lc3 compared to Lc0 and Lc1.

The DH (Figure 1A) remained homogeneous across all formulations, ranging from approximately 110 to 126 nm. Similarly, the PdI (Figure 1B) remained consistently low (0.125 to 0.142) regardless of cholesterol content, indicating a uniform and monodisperse vesicle population. The ZP (Figure 1C) was also unaffected by cholesterol incorporation, with all formulations showing a narrow, consistently negative surface charge between −44 and −47 mV.

### 2.2. Liposomes Under Different Stresses

#### 2.2.1. Impact of Mechanical and Mechanical-Thermal Stresses

**Figure 2:**
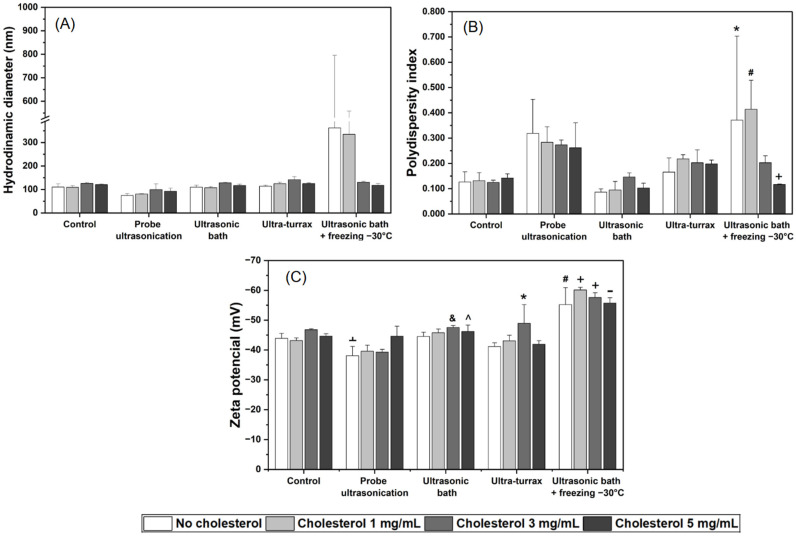
Effect of mechanical and mechanical–thermal stresses on the physicochemical properties of liposomes containing different concentrations of cholesterol (0, 1, 3, and 5 mg/mL). (**A**) Hydrodynamic diameter, (**B**) polydispersity index (PdI), and (**C**) Zeta potential (ZP) after exposure to the following stress conditions: control (no stress), probe ultrasonication, ultrasonic bath, ultra-turrax, and alternating cycles of ultrasonic bath + freezing at −30 °C. Data represent mean ± SD of three replicates. Statistical significance was determined using one-way ANOVA followed by Tukey’s post hoc test (*p* < 0.05). Symbols above the bars indicate significant differences according to the following scheme: (**B**): * (Lc0ub); # (Lc0c, Lc3c, Lc0ub, Lc1ub, Lc5ub); + (Lc3uf). (**C**): ﬩ (Lc3c); & (Lc0pu, Lc1pu, Lc3pu); ^ (Lc0pu); * (Lc0pu, Lc1pu, Lc3pu, Lc0ut); # (Lc0c, Lc1c, Lc3c, Lc5c, Lc0pu, Lc1pu, Lc3pu, Lc5pu, Lc0ub, Lc1ub, Lc5ub, Lc0ut, Lc1ut, Lc5ut); + (Lc0c, Lc1c, Lc3c, Lc5c, Lc0pu, Lc1pu, Lc3pu, Lc5pu, Lc0ub, Lc1ub, Lc3ub, Lc5ub, Lc0ut, Lc1ut, Lc3ut, Lc5ut); - (Lc0c, Lc1c, Lc3c, Lc5c, Lc0pu, Lc1pu, Lc3pu, Lc5pu, Lc0ub, Lc1ub, Lc3ub, Lc5ub, Lc0ut, Lc1ut, Lc5ut).

The impact of mechanical and combined mechanical–thermal stresses on the physicochemical stability of the liposomal formulations was strongly influenced by the specific type of stress applied (Figure 2 and Supplementary Table S2).

Isolated mechanical stresses (probe ultrasonication, ultrasonic bath, and ultra-turrax) did not induce significant changes (*p* > 0.05) in DH (Figure 2A). In contrast, the combined ultrasound–freezing stress resulted in a marked increase in DH, particularly for Lc0uf and Lc1uf, which increased from approximately 110 nm and 109 nm to approximately 362 nm and 334 nm, respectively.

The homogeneity of the system, evaluated by the PdI, was also affected by the combined stress (Figure 2B). PdI values increased from 0.127 and 0.132 to approximately 0.371 and 0.413 for Lc0uf and Lc1uf, respectively, while isolated mechanical stresses did not significantly compromise vesicle homogeneity.

The ZP, a parameter indicative of colloidal surface stability, remained unchanged following isolated mechanical stresses, demonstrating the maintenance of membrane integrity (Figure 2C). In contrast, the combined ultrasound–freezing stress induced significant alterations (*p* < 0.05) in ZP across all formulations, with the most pronounced effects in Lc1uf, whose values shifted from −43.1 mV to −60.1 mV, respectively.

No significant differences were observed among formulations with varying cholesterol contents when exposed to combined ultrasonic bath + freezing at −30 °C stress. Therefore, under the tested conditions, cholesterol did not provide a detectable protective effect in maintaining liposomal colloidal stability under isolated mechanical stress.

#### 2.2.2. Impact of Thermal Stress

**Figure 3:**
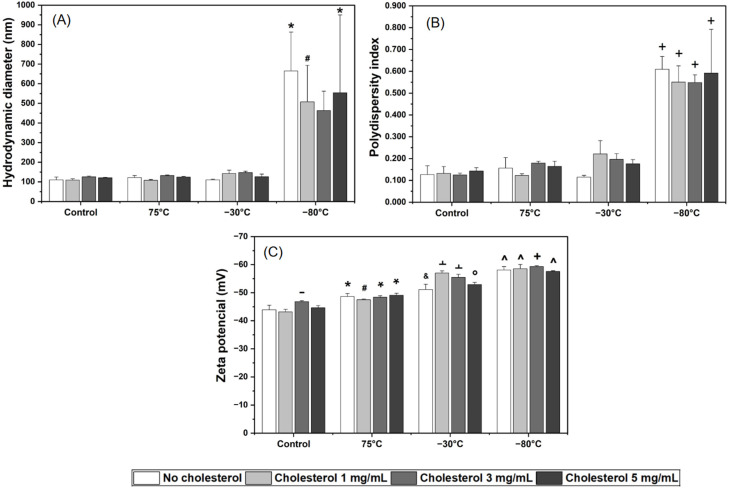
Effect of thermal stress on the physicochemical properties of liposomes containing different concentrations of cholesterol (0, 1, 3, and 5 mg/mL). (**A**) Hydrodynamic diameter, (**B**) polydispersity index (PdI), and (**C**) Zeta potential (ZP) after exposure to the following stress conditions: control (no stress), 75 °C, −30 °C, and −80 °C. Data represent mean ± SD of three replicates. Statistical significance was determined using one-way ANOVA followed by Tukey’s post hoc test (*p* < 0.05). Symbols above the bars indicate significant differences according to the following scheme: (**A**): * (Lc0c, Lc1c, Lc3c, Lc5c, Lc0t75, Lc1t75, Lc3t75, Lc5t75, Lc0t-30, Lc1t-30, Lc3t-30, Lc5t-30); # (Lc0c, Lc1c, Lc3c, Lc5c, Lc0t75, Lc1t75, Lc3t75, Lc5t75, Lc0t-30, Lc5t-30). (**B**): + (Lc0c, Lc1c, Lc3c, Lc5c, Lc0t75, Lc1t75, Lc3t75, Lc5t75, Lc0t-30, Lc1t-30, Lc3t-30, Lc5t-30). (**C**): - (Lc1c); * (Lc0c, Lc1c, Lc5c); # (Lc0c, Lc1c); & (Lc0c, Lc1c, Lc3c, Lc5c, Lc1t75); ﬩ (Lc0c, Lc1c, Lc3c, Lc5c, Lc0t75, Lc1t75, Lc3t75, Lc5t75, Lc0t-30); ° (Lc0c, Lc1c, Lc3c, Lc5c, Lc0t75, Lc1t75, Lc3t75, Lc5t75, Lc1t-30); ^ (Lc0c, Lc1c, Lc3c, Lc5c, Lc0t75, Lc1t75, Lc3t75, Lc5t75, Lc0t-30, Lc5t-30); + (Lc0c, Lc1c, Lc3c, Lc5c, Lc0t75, Lc1t75, Lc3t75, Lc5t75, Lc0t-30, Lc3t-30, Lc5t-30).

Thermal stress testing was conducted to simulate extreme temperature variations that liposomal formulations may experience during storage or transport. Liposomes were exposed to thermal stresses of 75 °C, −30 °C, and −80 °C, and subsequently evaluated for their physicochemical properties (Figure 3 and Supplementary Table S2).

Thermal exposure at a high temperature (75 °C) and moderate freezing (−30 °C) did not induce significant changes in DH (Figure 3A) or PdI (Figure 3B). In contrast, freezing at −80 °C proved to be a particularly severe stress condition, promoting an increase of more than fourfold in DH and fivefold in PdI for all formulations. The cholesterol-free formulation Lc0t-80 was the most affected formulation, reaching approximately 664 nm in DH and a PdI of 0.609, suggesting strong susceptibility to intense freezing.

With respect to ZP (Figure 3C), all thermal stresses led to significant increases compared to their respective non-stressed controls. Although statistical differences in ZP were observed among formulations subjected to freezing, no clear correlation was found with the presence or concentration of cholesterol.

#### 2.2.3. Impact of Chemical Stress

**Figure 4:**
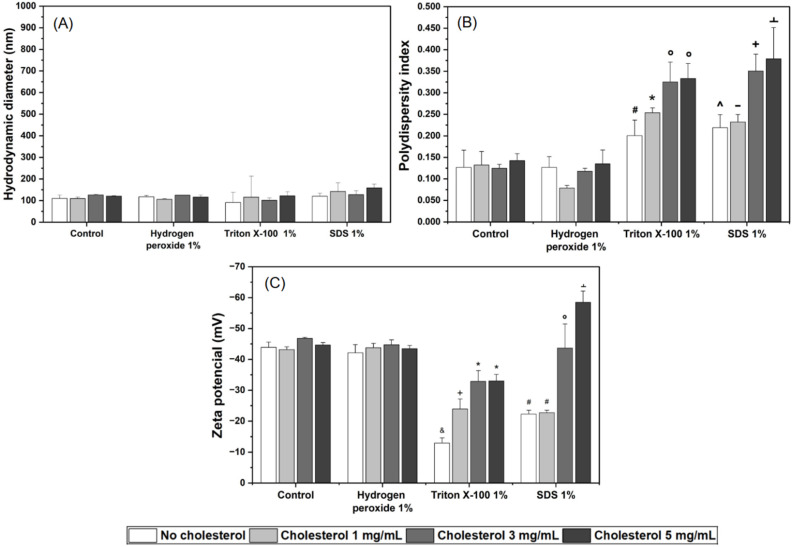
Effect of chemical stresses on the physicochemical properties of liposomes containing different concentrations of cholesterol (0, 1, 3, and 5 mg/mL). (**A**) Hydrodynamic diameter, (**B**) polydispersity index (PdI), and (**C**) Zeta potential (ZP) after exposure to the following stress conditions: control (no stress), hydrogen peroxide 1%, Triton X-100 1%, and SDS 1%. Data represent mean ± SD of three replicates. Statistical significance was determined using one-way ANOVA followed by Tukey’s post hoc test (*p* < 0.05). Symbols above the bars indicate significant differences according to the following scheme: (**B**): # (Lc1hp); * (Lc0c, Lc1c, Lc3c, Lc5c, Lc0hp, Lc1hp, Lc3hp, Lc5hp); ° (Lc0c, Lc1c, Lc3c, Lc5c, Lc0hp, Lc1hp, Lc3hp, Lc5hp, Lc0tx); ^ (Lc1hp, Lc3hp, Lc3tx, Lc5tx); - (Lc0c, Lc1c, Lc3c, Lc0hp, Lc1hp, Lc3hp, Lc5tx); + (Lc0c, Lc1c, Lc3c, Lc5c, Lc0hp, Lc1hp, Lc3hp, Lc5hp, Lc0tx, Lc0sds, Lc1sds); ﬩ (Lc0c, Lc1c, Lc3c, Lc5c, Lc0hp, Lc1hp, Lc3hp, Lc5hp, Lc0tx, Lc1tx, Lc0sds, Lc1sds). (**C**): & (Lc0c, Lc1c, Lc3c, Lc5c, Lc0hp, Lc1hp, Lc3hp, Lc5hp); + (Lc0c, Lc1c, Lc3c, Lc5c, Lc0hp, Lc1hp, Lc3hp, Lc5hp, Lc0tx); * (Lc0c, Lc1c, Lc3c, Lc5c, Lc0hp, Lc1hp, Lc3hp, Lc5hp, Lc0tx, Lc1tx); # (Lc0c, Lc1c, Lc3c, Lc5c, Lc0hp, Lc1hp, Lc3hp, Lc5hp, Lc0tx, Lc3tx, Lc5tx); ° (Lc0tx, Lc1tx, Lc3tx, Lc5tx, Lc0sds, Lc1sds); ﬩ (Lc0c, Lc1c, Lc3c, Lc5c, Lc0hp, Lc1hp, Lc3hp, Lc5hp, Lc0tx, Lc1tx, Lc3tx, Lc5tx, Lc0sds, Lc1sds, Lc3sds).

The effects of chemical stress on the physicochemical properties of the different formulations are shown in Figure 4 and Supplementary Table S2. No significant changes were observed in DH (Figure 4A) for any of the formulations subjected to chemical stress. Additionally, incubation with 1% hydrogen peroxide did not alter DH, PdI, or ZP, indicating that the formulations were relatively resistant to oxidation.

In contrast, significant changes in PdI (Figure 4B) and ZP (Figure 4C) were observed in formulations incubated with 1% Triton X-100 and 1% sodium dodecyl sulfate (SDS) surfactants. PdI increased more pronounced in formulations with higher cholesterol content (Lc3tx, Lc3sds, Lc5tx, and Lc5sds) than in those with low or no cholesterol (Lc0tx, Lc1tx, Lc0sds, and Lc1sds), for both Triton X-100 and SDS. For the Triton X-100 group, ZP showed a clear dependence on cholesterol content: Lc0tx exhibited the greatest reduction in surface charge compared to cholesterol-containing formulations. Specifically, Lc0tx and Lc1tx were more affected by the stress than Lc3tx and Lc5tx, which contained higher cholesterol concentrations. The SDS-treated group displayed distinct responses among the different formulations: Lc0sds and Lc1sds showed a decrease in ZP (reduced surface charge), while Lc5sds exhibited an increase in ZP.

### 2.3. Functional Response of Encapsulated Streptomycin

The antimicrobial activity of encapsulated streptomycin was evaluated after stress conditions—ultrasound–freezing cycles, freezing at −80 °C, and incubation with 1% SDS—against *Escherichia coli* and *Staphylococcus aureus*, to assess whether the functional integrity of the drug was preserved using the minimum inhibitory concentration (MIC) assay.

Regardless of cholesterol concentration, all liposomal formulations subjected to the same stress condition exhibited identical MIC and MBC values against both *Escherichia coli* and *Staphylococcus aureus*.

For *E. coli*, formulations stressed by freezing at −80 °C and by the combined cycle maintained an MIC of 50 µg/mL, identical to the non-stressed control and to free streptomycin exposed to the same stresses. This indicates that the antibiotic’s efficacy was not compromised by these physical stressors. Conversely, both liposomal and free streptomycin samples incubated with SDS completely lost their antimicrobial activity.

For *S. aureus*, all stressed liposomal formulations retained antimicrobial activity. Notably, SDS treatment, despite its membrane-disrupting properties, did not impair antibiotic activity. For *S. aureus*, instead, it resulted in a decrease in MIC (from 12.5 µg/mL to 5.625 µg/mL) and consequently enhanced antibiotic activity, an effect also observed for free streptomycin exposed to SDS. This suggests the possibility of an interaction related to SDS membranolytic properties or enhanced drug bioavailability in the presence of the anionic surfactant.

Minimum bactericidal concentration (MBC) results were consistent with the MIC profiles, confirming that the bactericidal activity of liposome-encapsulated streptomycin was preserved following exposure to the tested stresses.

## 3. Discussion

Based on the formulation and physicochemical characterization of streptomycin-loaded liposomes with different cholesterol contents, this study evaluates their stability under mechanical, thermal, and chemical stresses relevant to pharmaceutical processing and storage. While previous studies have focused on formulation or basic characterization, this work extends the analysis by examining how such stresses affect both liposomal stability and antimicrobial activity, providing a more application-oriented understanding of the relationship between lipid composition, stability, and functional performance.

### 3.1. Evaluation of the Effect of Cholesterol on DH, PdI, and ZP

Cholesterol is a steroid that, when incorporated into liposomal formulations, is inserted between phospholipid molecules to promote tighter molecular packing and increase bilayer rigidity. This effect often enhances vesicle stability, a crucial attribute for their application as DDS [22,28,29].

Although no universally defined cholesterol concentration exists to optimize stability and DDS performance, ratios of 1:1 and 2:1 (phospholipids/cholesterol) are the most frequently reported in the literature [27]. This molecule is known to induce various physicochemical changes in vesicles, but our findings suggest that in systems subjected to extrusion, the influence of cholesterol may be less pronounced or not universally applicable, as is typically reported in previous studies [30,31].

Additionally, changes in liposomal composition may significantly affect drug bioavailability and release behavior, highlighting the importance of experimental design approaches to optimize formulation parameters and better understand nanosystem stability [38,39].

The absence of significant changes in DH and PdI with increasing cholesterol concentrations (Figure 1A,B) contrasts with several studies documenting increases in vesicle size and polydispersity [29,31,32]. Cholesterol is typically associated with increased bilayer rigidity and restricted curvature, which could lead to an increase in DH [32,33]. However, this effect is strongly dependent on the preparation method. Therefore, the extrusion process played a central role in homogenizing vesicle size, even across different compositions.

Extrusion is a method widely used to reduce vesicle size, homogenize the population, and convert multilamellar liposomes into unilamellar vesicles. By forcing the formulation through a membrane with a defined pore size, it is possible to obtain DDS with similar DH and low PdI, regardless of initial compositional variations [34]. Our study demonstrates that, under standardized extrusion conditions, a highly homogeneous liposomal population in terms of size and distribution was obtained independently of cholesterol content, reinforcing the dominance of processing parameters over compositional variables.

Regarding ZP (Figure 1C), the negative values observed (between −42 and −47 mV) are an intrinsic characteristic of L-α-phosphatidylcholine-based liposomes. Despite being zwitterionic, the exposed phosphate group on the vesicle surface confers a net negative charge [35,40]. The electrical neutrality of cholesterol may explain the absence of significant interference in this parameter, as its incorporation does not modify the resultant surface charge. Moreover, ZP values with a magnitude greater than |40| mV across all formulations indicate excellent colloidal stability [41].

### 3.2. Stability Assessment Under Stress Conditions

The applied mechanical, thermal, and chemical stresses were deliberately chosen to represent pharmaceutical handling, storage, and processing conditions, while providing sufficient severity to discriminate formulation robustness. Physical stresses were selected to simulate mechanical stimulus release, shear, and cavitation forces encountered during physiological processes, such as blood circulation [42,43,44]. Heating at 75 °C was employed as an accelerated thermal stress to mimic rapid membrane destabilization and degradation pathways. Freezing at −30 °C reflects standard laboratory and short-term pharmaceutical storage, whereas freezing at −80 °C corresponds to long-term storage, transport, and biobanking conditions [25,45]. Oxidative stress mediated by hydrogen peroxide occurs naturally in inflammatory and injured tissues, bacterial infections, and cancer cell environments [46,47,48]. Although detergents are not physiologically present, endogenous agents such as bile salts, pulmonary phospholipids, and others exert analogous membrane insertion and destabilization effects, which are crucial for understanding liposome bilayer stability [49].

The variations observed in hydrodynamic diameter (DH), polydispersity index (PdI), and Zeta potential (ZP) provide essential insights into the mechanisms governing liposomal stability, which can be interpreted through the lens of Derjaguin–Landau–Verwey–Overbeek (DLVO) theory [50,51]. According to this theory, colloidal stability arises from the balance between attractive van der Waals forces and repulsive electrostatic interactions, determining the propensity for vesicle aggregation, fusion, or destabilization [50,52]. In this context, increases in DH and PdI reflect a reduction in the energy barrier between particles, indicating aggregation events or population heterogeneity, while ZP quantifies the magnitude of electrostatic repulsion responsible for the system’s kinetic stability [51,53,54]. High absolute ZP values (>30 mV) [41] contribute to maintaining stable dispersions even under external perturbations.

These observed stability trends not only reflect the formulation composition but also illustrate how applied stresses modulate interparticle interactions and energy barriers predicted by DLVO theory, thereby impacting the physicochemical stability and functional performance of streptomycin-loaded liposomes [51,53,54]

#### 3.2.1. Liposomes Under Mechanical Stress

The mechanical stresses applied in this study (probe ultrasonication, ultrasonic bath, and ultra-turrax homogenization) operate through distinct mechanisms and energy levels. Both probe ultrasonication and ultrasonic bath rely on cavitation, a process in which the formation and collapse of microbubbles generate shear forces, and shearing, promoting vesicle fragmentation and temporary lamellar permeability [55]; however, the probe delivers this energy more intensely and in a more localized manner. Ultra-turrax, in contrast, promotes mechanical shear through high-speed rotation [56].

Although shear-based techniques are explored as stimuli for controlled drug release [57], they are also routinely employed in liposome production to reduce size and polydispersity, as the applied energy promotes rearrangements within the lipid bilayer [42].

The liposomal formulations exhibited remarkable resilience to isolated mechanical stress conditions. While the literature reports that giant unilamellar vesicles (GUVs) exposed to ultrasound may undergo leakage and shrinkage [43], our results did not reveal significant changes in DH, PdI, or ZP following probe ultrasonication, ultrasonic bath, or ultra-turrax homogenization. This stability may be attributed to the dynamic and self-reorganizing nature of lipid bilayers, whose fluid and deformable structure can absorb mechanical energy and reorganize into stable nanosized vesicles [6,42,58,59] or even to the greater mechanical robustness conferred by the formation process itself (extrusion). Since these vesicles were molded under intense mechanical stress, even higher energy levels would be required to cause further significant structural changes [34,44]. In contrast, the combined ultrasound–freezing stress induced marked alterations in the formulations, particularly in Lc0 and Lc1. The sharp increase in DH (from approximately 110 nm to >360 nm) and PdI (from approximately 0.13 to >0.37), along with shifts in ZP (from approximately −43 mV to −55/−60 mV), strongly indicate freeze–thaw–induced vesicle fusion and membrane disruption. This profile suggests vesicle fusion and rupture events, consistent with reports that freeze–thaw cycles promote lipid rearrangements, ice-crystal formation, and vesicle collapse [23,60].

Although the ultrasonic bath alone did not alter physicochemical parameters, the significant difference in ZP between this group and the combined stress group reinforces that freezing, rather than ultrasound, was the dominant destabilizing factor. The subsequent ultrasonic exposure may have accelerated the thawing rate, thereby exacerbating bilayer disruption compared with slow thawing at room temperature [60].

Finally, the results did not demonstrate a clear influence of cholesterol on resistance to physical stress, even though literature frequently highlights its role in increasing lipid packing and membrane rigidity [29]. This suggests that the magnitude of mechanical–thermal damage exceeded the stabilizing capacity provided by cholesterol under the conditions tested.

#### 3.2.2. Liposomes Under Thermal Stress

It is known that increasing temperature raises the random kinetic energy of the DDS, thereby increasing collision frequency and promoting vesicle aggregation [61]. However, heating to 75 °C, as well as moderate freezing at −30 °C, did not result in relevant changes in DH or PdI, suggesting that the formulations possess moderate thermal resilience. Studies, such as that by Žiga Pandur et al., observed that GUVs exhibited alterations at temperatures above 80 °C [43], and other studies show the change to be buffer-dependent [45].

Freezing can promote ice-crystal formation, induce aggregation, and cause osmotic damage. Therefore, the use of cryoprotectants is generally recommended for samples stored under freezing conditions or subject to cooling stress [23,60]. In this context, exposure to −80 °C led to a marked increase in DH, PdI, and ZP, which may be attributed to structural disruption of the liposomes caused by the freezing process. The freezing rate and the phospholipid composition are known to influence the extent of liposomal alterations [60].

The physicochemical modifications observed after thermal stress are directly related to the thermotropic behavior of phospholipids. Due to its heterogeneous composition, soybean phosphatidylcholine exhibits a broad phase transition temperature range (Tm approximately 20 °C for PC) [25]. During freezing, the different lipid components undergo phase transitions at distinct temperatures, promoting structural rearrangements that affect DH, PdI, and ZP [60,61]. The phase transition eventually reduces bilayer stability, rendering it more susceptible to damage. Thermal stresses directly influence the fluidity, compressibility, and stiffness of the lipid bilayer, particularly when navigating transitions between gel and liquid phases [23,25]. These alterations facilitate vesicular fusion and structural reorganization during freezing, as evidenced by the significant variations in DH, PdI and ZP. Cholesterol acts as a stabilizer by broadening the Tm and inducing the formation of a liquid-ordered phase. This structural arrangement increases molecular packing and bilayer rigidity, reducing permeability and improving resistance to thermal variations [60,62]. Accordingly, formulations containing cholesterol exhibited slightly attenuated variations in physicochemical parameters following freezing compared with cholesterol-free formulations [62]. Hence, temperature variations were able to modulate the bilayer fluidity and rigidity of the liposomes, leading to distinct responses depending on the cholesterol concentration, as also reported by Sułkowski [25].

#### 3.2.3. Liposomes Under Chemical Stress

Free radicals’ generation is an efficient mechanism for vesicle destabilization, mediated by phospholipid oxidation that induces pore formation and bilayer disintegration [43,63]. Although hydrogen peroxide (H_2_O_2_), a byproduct of aerobic metabolism, can oxidize phospholipids [64], it did not induce any relevant changes in DH, PdI, or ZP under the tested conditions. This resistance may indicate that the concentration used was below the critical threshold required for destabilization, despite reports describing liposomes responsive to low H_2_O_2_ concentrations, such as 0.1% [65] and in tumor cell-reducing environment models [46].

In contrast, the surfactants Triton X-100 and SDS significantly impacted liposome integrity. Both induced increases in PdI and alterations in ZP, indicating bilayer disorganization due to the destabilization and decompression caused by the insertion of surfactants into the lipid bilayer [40,43]. Triton X-100, a nonionic surfactant, interacts with the hydrophobic region, fluidizing the membrane and promoting vesicle disruption upon reaching its critical micelle concentration, in addition to partially reducing intervesicular electrostatic repulsion [43,66]. SDS promoted bilayer disruption, evidenced by a marked increase in PdI and marked changes in ZP. The insertion of this anionic surfactant into the bilayer, partially solubilizing the membrane, potentially alters the distribution of surface charges, resulting in responses dependent on lipid composition [65,67].

Liposome destabilization is evidenced by the alteration of the Zeta potential (ZP), which may be related to a reduction in electrostatic repulsion between vesicles, favoring fusion processes. Additionally, the increased permeability of the bilayer and the consequent heterogeneity of sizes result in an increase in the PdI [51].

The distinct responses to chemical stress among formulations can be associated with cholesterol. Cholesterol modulates phospholipid organization by increasing bilayer rigidity and reducing permeability. This effect likely delays surfactant insertion, thereby attenuating abrupt alterations and conferring greater resilience according to previous reports [22,68].

The stability of the formulations under physical stress, which was not observed under chemical stress, suggests that, in addition to the DLVO theory and the mechanical strength formed by the extrusion, repulsive hydration forces [51,69] as supplementary stabilizing mechanisms. This hydrophilic character creates an energy barrier that confers mechanical robustness against physical impacts [69]. However, such protection is ineffective against chemical stress, since surfactants disrupt the hydrophilic interface and promote the solubilization of the lipid bilayer.

The comparable MIC and MBC values observed between stressed liposomal formulations and free streptomycin indicate that sufficient antibiotic release occurred to maintain full bioactivity, likely as a consequence of stress-induced vesicle disruption without compromising drug integrity.

### 3.3. Antimicrobial Activity of Encapsulated Streptomycin

The response to 1% SDS stress was dependent on bacterial species. For *S. aureus* (Gram-positive), SDS potentiated the action of streptomycin, reducing the MIC. This effect may be attributed to the surfactant’s synergistic action on the single cellular membrane, facilitating the internalization of the antibiotic at its intracellular site of action [51].

In contrast, no MIC was identified for *E. coli* (Gram-negative), indicating a loss of antimicrobial activity at this concentration. The proposed hypothesis is that an inactive complex forms between the anionic surfactant (SDS) and cationic functional groups of the streptomycin molecule as previously reported [70]. The species-dependent nature of this effect may relate to structural differences in the bacterial cell wall, which influence the accessibility and interaction of the complex with the microbial target; however, this mechanism remains not fully elucidated.

Furthermore, the similar antimicrobial performance among formulations with different cholesterol contents indicates that, although cholesterol influences physicochemical stability, it does not critically influence the final efficacy of streptomycin. This supports the premise that for hydrophilic antibiotics encapsulated in the aqueous core, moderate modifications in bilayer composition do not impact their diffusion into the medium [22]. Instead, cholesterol is more likely to modulate stress-induced release kinetics and membrane integrity over time. However, this contrasts with studies demonstrating that cholesterol enhances the antimicrobial efficiency of liposomes containing antimicrobials; for instance, concentrations of 20% and 50% cholesterol proportionally increased antibacterial effects against Staphylococcus epidermidis, with bacterial growth inhibition showing a linear relationship to cholesterol content [21]. Similarly, liposomes with cholesterol have been shown to act against biofilms, further highlighting their role in potentiating antimicrobial activity in certain contexts [36,37].

## 4. Materials and Methods

### 4.1. Materials

Ultrapure Type I water (Gehaka, Sao Paulo, Brazil); pure bacteriological agar-agar powder CAS 9002-18-0 (Vetec, Sigma-Aldrich, Duque de Caxias, Brazil); streptomycin sulfate purex CAS 3810-74-0 (Inlab, Diadema, Brazil); cholesterol 99% (Sigma-Aldrich, St. Louis, MO, USA); chloroform P.A. 99% (J.T. Baker, Mexico City, Mexico); L-α-phosphatidylcholine from soybean type II 14–29% choline (PC; Sigma-Aldrich, Steinheim, Germany); Mueller–Hinton broth (TM Media, Rajasthan, India); 2,3,5-triphenyltetrazolium chloride (TTC; NEON, Sao Paulo, Brazil); phosphate-buffered saline (PBS; Amresco, Solon, OH, USA); hydrogen peroxide 35% P.A. (NEON, Suzano, Brazil); ultrapure sodium dodecyl sulfate 99.5% (SDS; Invitrogen, Carlsbad, CA, USA); Triton X-100 pure 98% (ProQuimios, Rio de Janeiro, Brazil).

### 4.2. Liposome Preparation

Liposomes were produced using the classic lipid film hydration method described by Bangham et al. [71], followed by manual extrusion processes. Formulations contained soybean L-α-phosphatidylcholine (PC) at a final lipid concentration of 10 mg/mL, combined with 0, 1, 3, or 5 mg/mL of cholesterol (CHO). Stock solutions of PC (20 mg/mL) and cholesterol (10 mg/mL), both dissolved in chloroform P.A., were transferred to 100 mL round-bottom flasks according to the desired lipid ratios. The final volume was adjusted to 10 mL with chloroform. Lipid films were formed by microprocessed rotary evaporation under controlled conditions (Quimis, Sao Paulo, Brazil) for 1 h at 40 °C, using an ultra-thermostatic condenser bath at 10 °C and 300 mmHg vacuum pressure, to ensure complete solvent removal and uniform lipid film formation.

The dry lipid films were hydrated with 10 mL of streptomycin sulfate solution (500 µg/mL in PBS), maintaining a 1:1 ratio between the initial lipid solution and the hydration phase. Hydration was performed under vigorous vortex mixing (KASVI, Quincy, MA, USA) at maximum speed for 10 min, producing relatively homogeneous multilamellar liposomal suspensions. Formulations containing 0, 1, 3, and 5 mg/mL of cholesterol were designated Lc0, Lc1, Lc3, and Lc5, respectively.

To obtain nanosized unilamellar vesicles, the suspensions underwent manual extrusion using a Mini-Extruder Set (Avanti Polar Lipids, Alabaster, AL, USA). For each batch, 1 mL of liposomal suspension was loaded into a Hamilton syringe and repeatedly passed through a 100 nm pore polycarbonate membrane mounted on the extruder. Each formulation underwent exactly 13 extrusion cycles to ensure size homogeneity. The polycarbonate membranes were pre-hydrated and rinsed with ultrapure water between samples to minimize material retention. After extrusion, liposomal formulations were stored at 4 °C (Bosch, Gerlingen, Germany) until further characterization.

### 4.3. Stress Test Methodologies

For each liposomal formulation (Lc0, Lc1, Lc3, and Lc5), 200 µL aliquots were prepared in triplicate for the assays, which were categorized into mechanical, thermal, combined mechanical–thermal, and chemical stresses. All formulations were subjected to the ten stress conditions described below. A non-stressed control group was maintained at 4 °C until characterization. Free streptomycin (500 µg/mL) was also used as a control group (200 µL) and exposed to the same stress conditions for subsequent functional assessment.

#### 4.3.1. Mechanical Stress Test

Mechanical stress was applied using three distinct procedures. In the first, samples were exposed to probe ultrasonication using an ultrasonic processor (Ecosonics, Indaiatuba, Brazil) operating at 20% of the maximum potential, in alternating cycles of 30 s on and 30 s off for a total of 5 min. Samples were kept on ice throughout the process, and the probe was cleaned between treatments. In the second procedure, liposomes were subjected to high-shear mechanical disruption using a modified Dremel-based ultra-turrax system (Marconi, Sao Paulo, Brazil) at speed level 2 (approximately 14,600 rpm), also in 30 s pulses alternating with 30 s intervals for a total of 5 min, with cooling on ice to prevent temperature rise. The third mechanical stress involved placing the samples in an ultrasonic bath (Quimis, Brazil) operating at 37 kHz for 45 min under ice bath conditions.

#### 4.3.2. Thermal Stress Test

Thermal stress was assessed under three temperature conditions. In the first, samples were frozen at −80 °C for 45 min in an ultra-freezer (TDE Series, Thermo Scientific, Waltham, MA, USA). In the second, aliquots were heated at 75 °C in a water bath (SolidSteel, Piracicaba, Brazil) for 45 min. In the third, samples were frozen (Eletrolux, Sao Paulo, Brazil) at −30 °C for 45 min. Following each thermal exposure, samples were allowed to equilibrate to room temperature before characterization by dynamic light scattering (DLS).

#### 4.3.3. Combined Mechanical and Thermal Stress

To evaluate the combined effect of mechanical and thermal stress, an additional group of samples was subjected to alternating cycles consisting of 15 min in an ultrasonic bath (37 kHz, ice bath) (Quimis, Brazil) followed by 15 min at −30 °C freezing (Eletrolux, Brazil). Three consecutive cycles were completed, resulting in a total exposure of 45 min to each stress type. After the final cycle, samples were equilibrated to room temperature for analysis.

#### 4.3.4. Chemical Stress Test

Chemical stress was induced by exposing the liposomes to oxidizing and surfactant agents. Hydrogen peroxide was added to a group of aliquots to a final concentration of 1% (*v*/*v*). In separate treatments, Triton X-100 and SDS were also added to reach a final concentration of 1% (*v*/*v*). After 30 min of exposure to these agents, all samples were diluted 1:20 in ultrapure water prior to DLS analysis, as described in Section 4.5.

### 4.4. Minimum Inhibitory Concentration Test

The liposomal formulations selected for the MIC assays were those subjected to combined physical stress (ultrasound–freezing cycles), extreme thermal stress at −80 °C freezing, and chemical stress at 1% SDS. All four formulations with different cholesterol contents (Lc0, Lc1, Lc3, and Lc5) were evaluated. The antimicrobial activity of encapsulated streptomycin was assessed against *Escherichia coli* and *Staphylococcus aureus* after each stress condition.

In 96-well U-bottom microtiter plates, 40 µL of each liposomal formulation (in triplicate) was serially diluted in Mueller–Hinton (MH) broth to obtain streptomycin concentrations ranging from 50 to 0.78 µg/mL in a final well volume of 200 µL, except for liposomes incubated with SDS. Since the addition of detergent caused dilution, the test range for these samples was 45 to 0.703 µg/mL. These concentrations were calculated based on an initial encapsulated streptomycin concentration of 500 µg/mL in the liposomal preparations.

The antimicrobial assays employed *Staphylococcus aureus* (ATCC^®^ 25923) and *Escherichia coli* (ATCC^®^ 8739). Bacteria were recovered from isolated colonies grown overnight on Mueller–Hinton Agar (MHA) and subsequently incubated in MH broth at 35 °C under 120 rpm shaker agitation (Lucadema, São José do Rio Preto, Brazil). Bacterial density was quantified by optical density at 600 nm (OD_600_) using a BioPhotometer (Eppendorf, Hamburg, Germany) and adjusted to 0.05 AU, corresponding to approximately 1.5 × 10^7^ CFU/mL. The bacterial suspension was then added to the wells to achieve a final inoculum of 5 × 10^5^ CFU/mL. Plates were incubated at 35 °C in a bacteriological oven (Lucadema, Brasil).

After 22 h of incubation, 20 µL of 0.5% 2,3,5-triphenyltetrazolium chloride (TTC) were added to each well, followed by an additional 2 h incubation under the same conditions. Colorimetric changes in the medium were used to determine MIC values, based on TTC reduction by metabolically active bacteria.

To determine the minimum bactericidal concentration (MBC), 10 µL from each well (in triplicate) were inoculated onto MHA plates and incubated at 35 °C to assess colony growth.

### 4.5. Dynamic Light Scattering and Zeta Potential

The hydrodynamic diameter (DH), polydispersity index (PdI), and Zeta potential (ZP) of the liposomal formulations were determined by dynamic light scattering (DLS) and electrophoretic mobility measurements. Prior to analysis, all samples were diluted 1:20 by mixing 50 µL of liposomal suspension with 950 µL of ultrapure water. A total volume of 950 µL of the diluted sample was transferred to a disposable capillary cell (DTS 1070, Malvern Panalytical, Worcestershire, West Midlands, UK) for measurement. All characterizations were performed on the same day that the stress tests were conducted to avoid storage-related variability.

Analyses were carried out using a ZetaSizer Nano ZS (Malvern Panalytical, Worcestershire, UK) equipped with a 633 nm, 4 mW He–Ne laser operating at a 173° backscatter detection angle. Each sample underwent three consecutive measurements at 25 °C under automatic mode, and the results were expressed as arithmetic mean ± standard deviation (SD).

### 4.6. Statistical Analysis

For each formulation, three independent replicates were analyzed, with measurements performed in triplicate by DLS. Technical triplicates were averaged and used as a single value for statistical analysis. The data were reported as mean ± standard deviation (SD). Statistical analyses were performed using one-way analysis of variance (ANOVA) followed by Tukey’s post hoc test to determine differences among groups, with a significance threshold of *p* < 0.05. All statistical procedures were conducted using PAST v3.26 [72].

One-way ANOVA followed by Tukey’s post hoc test was performed separately for each stress category (mechanical, thermal, chemical, and combined), encompassing all formulations and conditions within that category. This global comparison strategy controls the family-wise error rate across multiple groups, providing a conservative assessment that prioritizes robust differences while minimizing false positives.

## 5. Conclusions

This study demonstrates that streptomycin-loaded liposomes composed of soybean phosphatidylcholine and varying cholesterol content exhibit remarkable stability under most isolated mechanical, thermal, and chemical stresses relevant to pharmaceutical processing, transport, and storage. Isolated mechanical stresses (ultrasonication, probe ultrasonication, or high-shear homogenization) and moderate thermal conditions (75 °C or freezing at −30 °C) induced no significant changes in DH, PdI, or ZP, indicating that bilayer integrity and colloidal stability were preserved. In contrast, combined stresses, particularly repeated ultrasound–freezing cycles and deep freezing at −80 °C, triggered vesicle fusion and aggregation, with the most pronounced effects observed in cholesterol-free formulations; for instance, combined ultrasound–freezing increased DH from ~110 nm to 362 nm (Lc0uf) and from 109 nm to 334 nm (Lc1uf), with PdI rising from ~0.13 to 0.37–0.41 and a ZP shift in Lc1uf from −43.1 mV to −60.1 mV, while −80 °C freezing led to over fourfold DH increases (e.g., Lc0t−80 reaching ~664 nm) and fivefold PdI increases (up to 0.609).

Cholesterol exerted a modulatory role in liposome stability, but its protective effect was stress-specific: it significantly enhanced resistance to chemical disruption by surfactants (Triton X-100 and SDS), where 1% concentrations altered PdI and ZP in a cholesterol-dependent manner, and partially mitigated damage from extreme freezing (−80° C), yet it offered no detectable protection against purely mechanical or combined mechanical–thermal stresses under the conditions tested.

Importantly, the antimicrobial activity of encapsulated streptomycin was fully preserved after exposure to all physical stresses that maintained vesicle integrity. Even after severe destabilizing conditions (−80 °C freezing or ultrasound–freezing cycles), the released antibiotic retained equivalent efficacy to non-stressed controls against both *E. coli* and *S. aureus*, with MIC values of 50 µg/mL for E. coli post-freezing or combined stress. The unexpected species-dependent interaction with SDS, complete loss of activity against *E. coli* but enhanced potency against *S. aureus* (MIC reduced from 12.5 to 5.625 µg/mL), highlights the complex interplay between surfactant, antibiotic, and bacterial cell wall structure and warrants further mechanistic investigation.

In summary, these robust liposomal formulations protect streptomycin bioactivity under a wide range of realistic stress conditions, with cholesterol acting as a valuable modulator of membrane rigidity and chemical resilience. The results support their potential as stable, effective platforms for combating antimicrobial resistance and open avenues for designing stimulus-responsive systems that exploit controlled destabilization (e.g., surfactant- or extreme cold-triggered release) for targeted antimicrobial delivery. This work addresses a key gap by revealing stress-specific cholesterol modulation and preserved antibiotic efficacy post-destabilization, providing foundational insights for optimizing liposomal DDS in pharmaceutical applications.

Future studies employing molecular dynamics simulations, advanced spectroscopic techniques, or bilayer permeability assays could provide deeper insight into the molecular mechanisms underlying these stress responses and support the rational design of liposomal systems optimized either for maximal stability or for controlled, stimulus-triggered drug release.

## Figures and Tables

**Table 1:** Nomenclature and description of liposomal formulations and stress conditions evaluated in this study ^1^.

Formulation Code	Cholesterol (mg/mL)	Stress Abbreviation	Stress Condition
Lc0c, Lc1c, Lc3c, Lc5c	0, 1, 3, 5	c	Control
Lc0pu, Lc1pu, Lc3pu, Lc5pu	0, 1, 3, 5	pu	Probe ultrasonication
Lc0ub, Lc1ub, Lc3ub, Lc5ub	0, 1, 3, 5	ub	Ultrasonic bath
Lc0ut, Lc1ut, Lc3ut, Lc5ut	0, 1, 3, 5	ut	Ultra-turrax
Lc0uf, Lc1uf, Lc3uf, Lc5uf	0, 1, 3, 5	uf	Ultrasonic bath + freezing
Lc0t75, Lc1t75, Lc3t75, Lc5t75	0, 1, 3, 5	t75	75 °C
Lc0t-30, Lc1t-30, Lc3t-30, Lc5t-30	0, 1, 3, 5	t-30	−30 °C
Lc0t-80, Lc1t-80, Lc3t-80, Lc5t-80	0, 1, 3, 5	t-80	−80 °C
Lc0hp, Lc1hp, Lc3hp, Lc5hp	0, 1, 3, 5	hp	Hydrogen peroxide
Lc0tx, Lc1tx, Lc3tx, Lc5tx	0, 1, 3, 5	tx	Triton X
Lc0sds, Lc1sds, Lc3sds, Lc5sds	0, 1, 3, 5	sds	SDS

^1^ The full nomenclature for each sample is a combination of the liposomal formulation code (Lc0, Lc1, Lc3, or Lc5, corresponding to cholesterol content of c0, c1, c3, or c5 mg/mL, respectively) and the stress abbreviation.

## Data Availability

The original contributions presented in the study are included in the article, further inquiries can be directed to the corresponding author.

## Supplementary Materials

The following supporting information can be downloaded at: https://www.mdpi.com/article/10.3390/ph19030366/s1, Figure S1: Liposome size distribution profiles of (A) Lc0, (B) Lc1, (C) Lc3, and (D) Lc5 formulations, with (E) overlay of all four distributions for comparative analysis; Figure S2: Zeta potential distribution profiles of liposomes: (A) Lc0, (B) Lc1, (C) Lc3, and (D) Lc5 formulations. The three curves in each panel correspond to triplicate measurements of the same sample; Table S1: Nomenclature and description of liposomal formulations and stress conditions evaluated in this study ^1^; Table S2: Mean values of hydrodynamic diameter (DH), polydispersity index (PdI), and Zeta potential (ZP) for all liposomal formulations under the different stress conditions ^1^.

## Abbreviations

The following abbreviations are used in this manuscript:

°C	Degrees Celsius
µg	Microgram
µL	Microliter
CHO	Cholesterol
CFU	Colony-forming unit
DDS	Drug delivery system
DH	Hydrodynamic diameter
DLS	Dynamic light scattering
GUV	Giant unilamellar vesicle
kHz	Kilohertz
MBC	Minimum bactericidal concentration
MIC	Minimum inhibitory concentration
mg	Milligram
mL	Milliliter
nm	Nanometer
PC	L-α-phosphatidylcholine
PdI	Polydispersity index
rpm	Revolutions per minute
SDS	Sodium dodecyl sulfate
SD	Standard deviation
Tm	Phase transition temperature
USA	United States of America
ZP	Zeta potential
