# Supplementary material for: Engineering Stability: Cholesterol-Modulated Liposome Response to Physical and Chemical Stressors for Enhanced Antimicrobial Activity
Source: Pharmaceuticals (Basel). 2026 Feb 26;19(3):366. doi: 10.3390/ph19030366 (PMC13029226; doi:10.3390/ph19030366)
Supplement: Supplementary file 1 [file pharmaceuticals-19-00366-s001.zip › pharmaceuticals-4072440-supplementary.pdf]

pharmaceuticals

Supplementary Table S1. Nomenclature and description of liposomal formulations and stress conditions evaluated in this study [1]

Formulation code	Cholesterol content (mg/mL)	Stress abbreviation	Stress condition	Description of stressor
Lc0c	0	c	Control	No stress applied
Lc1c	1	c	Control	No stress applied
Lc3c	3	c	Control	No stress applied
Lc5c	5	c	Control	No stress applied
Lc0pu	0	pu	Probe ultrasonication	5 min (30 s on/off)
Lc1pu	1	pu	Probe ultrasonication	5 min (30 s on/off)
Lc3pu	3	pu	Probe ultrasonication	5 min (30 s on/off)
Lc5pu	5	pu	Probe ultrasonication	5 min (30 s on/off)
Lc0ub	0	ub	Ultrasonic bath	45 min
Lc1ub	1	ub	Ultrasonic bath	45 min
Lc3ub	3	ub	Ultrasonic bath	45 min
Lc5ub	5	ub	Ultrasonic bath	45 min
Lc0ut	0	ut	Ultra-turrax	5 min (30 s on/off)
Lc1ut	1	ut	Ultra-turrax	5 min (30 s on/off)
Lc3ut	3	ut	Ultra-turrax	5 min (30 s on/off)
Lc5ut	5	ut	Ultra-turrax	5 min (30 s on/off)
Lc0uf	0	uf	Ultrasonic bath + freezing	15 min ub –30°C
Lc1uf	1	uf	Ultrasonic bath + freezing	15 min ub –30°C
Lc3uf	3	uf	Ultrasonic bath + freezing	15 min ub –30°C
Lc5uf	5	uf	Ultrasonic bath + freezing	15 min ub –30°C
Lc0t75	0	t75	75°C	75°C 45 min
Lc1t75	1	t75	75°C	75°C 45 min
Lc3t75	3	t75	75°C	75°C 45 min
Lc5t75	5	t75	75°C	75°C 45 min
Lc0t-30	0	t-30	-30°C	–30°C 45 min
Lc1t-30	1	t-30	-30°C	–30°C 45 min
Lc3t-30	3	t-30	-30°C	–30°C 45 min
Lc5t-30	5	t-30	-30°C	–30°C 45 min
Lc0t-80	0	t-80	-80°C	–80°C 45 min
Lc1t-80	1	t-80	-80°C	–80°C 45 min
Lc3t-80	3	t-80	-80°C	–80°C 45 min
Lc5t-80	5	t-80	-80°C	–80°C 45 min
Lc0hp	0	hp	Hydrogen peroxyde	1% H_2O_2 30 min
Lc1hp	1	hp	Hydrogen peroxyde	1% H_2O_2 30 min
Lc3hp	3	hp	Hydrogen peroxyde	1% H_2O_2 30 min
Lc5hp	5	hp	Hydrogen peroxyde	1% H_2O_2 30 min
Lc0tx	0	tx	Triton X	1% Triton X-100
Lc1tx	1	tx	Triton X	1% Triton X-100
Lc3tx	3	tx	Triton X	1% Triton X-100
Lc5tx	5	tx	Triton X	1% Triton X-100
Lc0sds	0	sds	SDS	1% SDS
Lc1sds	1	sds	SDS	1% SDS
Lc3sds	3	sds	SDS	1% SDS
Lc5sds	5	sds	SDS	1% SDS

[1] The full nomenclature for each sample is a combination of the formulation code and the stress abbreviation.

Supplementary Table S2. Mean values of hydrodynamic diameter (DH), polydispersity index (PdI), and Zeta potential (ZP) for all liposomal formulations under the different stress conditions [1].

Name	DH (nm)	PdI	ZP (mV)	Stress
Lc0c	110.03	0.127	-43.88	Control
Lc1c	109.20	0.132	-43.16	Control
Lc3c	125.71	0.125	-46.79	Control
Lc5c	120.48	0.142	-44.63	Control
Lc0pu	74.07	0.319	-38.09	Probe ultrasonication
Lc1pu	80.81	0.283	-39.57	Probe ultrasonication
Lc3pu	99.48	0.273	-39.27	Probe ultrasonication
Lc5pu	92.35	0.262	-44.59	Probe ultrasonication
Lc0ub	109.52	0.086	-44.49	Ultrasonic bath
Lc1ub	107.20	0.095	-45.76	Ultrasonic bath
Lc3ub	127.68	0.146	-47.52	Ultrasonic bath
Lc5ub	116.67	0.103	-46.19	Ultrasonic bath
Lc0ut	113.72	0.166	-41.14	Ultra-turrax
Lc1ut	124.52	0.218	-43.09	Ultra-turrax
Lc3ut	141.31	0.202	-48.90	Ultra-turrax
Lc5ut	125.47	0.198	-41.88	Ultra-turrax
Lc0uf	362.10	0.371	-55.20	Ultrasonic bath + freezing
Lc1uf	334.57	0.414	-60.09	Ultrasonic bath + freezing
Lc3uf	130.76	0.203	-57.59	Ultrasonic bath + freezing
Lc5uf	117.49	0.116	-55.70	Ultrasonic bath + freezing
Lc0t75	121.76	0.156	-48.62	75°C
Lc1t75	107.54	0.122	-47.48	75°C
Lc3t75	131.91	0.179	-48.39	75°C
Lc5t75	124.21	0.164	-49.07	75°C
Lc0t-30	110.23	0.115	-48.62	-30°C
Lc1t-30	142.20	0.221	-47.48	-30°C
Lc3t-30	147.18	0.196	-48.39	-30°C
Lc5t-30	126.41	0.176	-49.07	-30°C
Lc0t-80	664.52	0.609	-58.13	-80°C
Lc1t-80	507.92	0.550	-58.56	-80°C
Lc3t-80	462.66	0.548	-59.27	-80°C
Lc5t-80	553.60	0.592	-57.58	-80°C
Lc0hp	117.23	0.126	-42.11	Hydrogen peroxyde
Lc1hp	105.43	0.079	-43.77	Hydrogen peroxyde
Lc3hp	124.54	0.118	-44.74	Hydrogen peroxyde
Lc5hp	115.84	0.135	-43.43	Hydrogen peroxyde
Lc0tx	90.98	0.200	-12.93	Triton X
Lc1tx	115.58	0.254	-23.91	Triton X
Lc3tx	100.78	0.325	-32.84	Triton X
Lc5tx	121.66	0.333	-32.99	Triton X
Lc0sds	119.93	0.219	-22.26	SDS
Lc1sds	142.13	0.232	-22.70	SDS
Lc3sds	127.47	0.351	-43.64	SDS
Lc5sds	158.50	0.379	-58.44	SDS

[1] The table presents the complete physicochemical dataset (mean values) obtained from dynamic light scattering (DLS) and electrophoretic mobility measurements. Formulations are labeled according to their cholesterol content (Lc0: 0 mg/mL; Lc1: 1 mg/mL; Lc3: 3 mg/mL; Lc5: 5 mg/mL) and the applied stress condition (see Table 1 in the main text for abbreviations). Each value represents the mean of three independent replicates. Standard deviations are omitted for clarity in the table but were used for all statistical analyses presented in the figures.

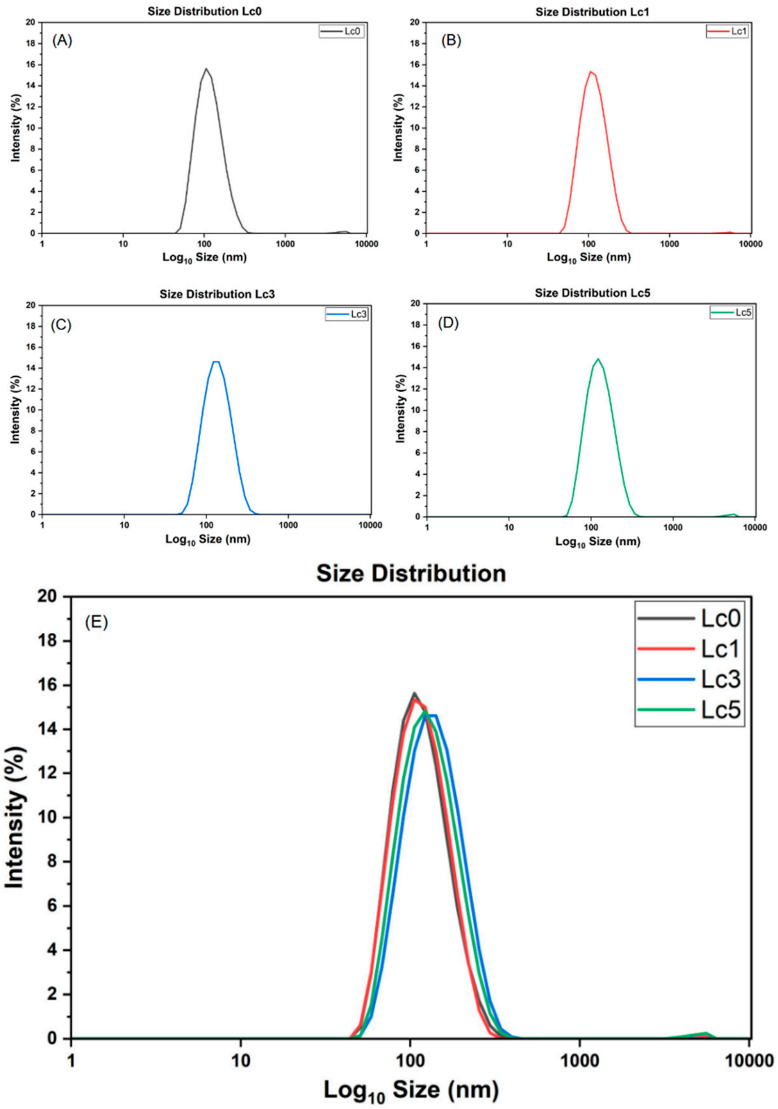

Supplementary Figure S1. Liposome size distribution profiles of (A) Lc0, (B) Lc1, (C) Lc3, and (D) Lc5 formulations, with (E) overlay of all four distributions for comparative analysis.

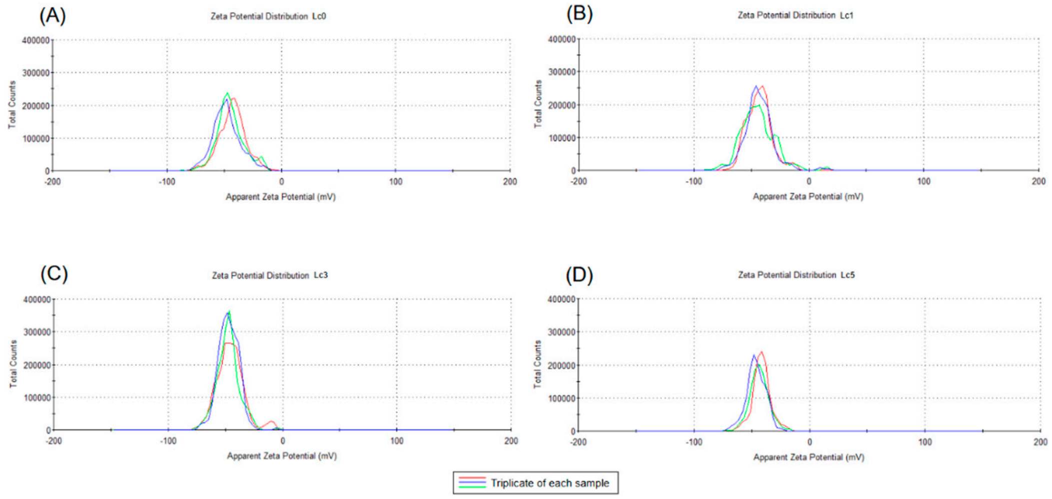

Supplementary Figure S2. Zeta potential distribution profiles of liposomes: (A) Lc0, (B) Lc1, (C) Lc3, and (D) Lc5 formulations. The three curves in each panel correspond to triplicate measurements of the same sample.
